# Factors Affecting Patient Portal Use Among Low-Income Pregnant Women: Mixed-Methods Pilot Study

**DOI:** 10.2196/formative.5322

**Published:** 2018-03-22

**Authors:** Juhee Kim, Holly Mathews, Lindsay M Cortright, Xiaoming Zeng, Edward Newton

**Affiliations:** ^1^ Department of Public Health Brody School of Medicine East Carolina University Greenville, NC United States; ^2^ Department of Anthropology East Carolina University Greenville, NC United States; ^3^ Department of Pediatrics East Carolina University Greenville, NC United States; ^4^ Department of Health Information and Management East Carolina University Greenville, NC United States; ^5^ Department of Obstetrics and Gynecology Brody School of Medicine East Carolina University Greenville, NC United States

**Keywords:** patient portals, digital divide, pregnancy, poverty, health literacy

## Abstract

**Background:**

Patient portals offer patients personalized and secure Web access to their medical information and enable patients to manage their health care online. However, there is a lack of information about patient acceptance and use of patient portals among low-income pregnant women.

**Objective:**

This formative research aims to assess the potential of a patient portal, MyChart, for improving prenatal health care and pregnancy outcomes, and identify the barriers and facilitators of MyChart use among low-income pregnant women.

**Methods:**

A mixed-methods study was conducted with a convenience sample of 18 low-income pregnant women comprising low- and high-risk patients enrolled in a prenatal clinic in eastern North Carolina. MyChart use, patient demographics, and pregnancy information were collected by reviewing electronic medical charts. Health literacy was measured. Reported use and attitudes toward MyChart were collected using a semi-structured interview.

**Results:**

Although 39% (7/18) of participants interviewed signed up for MyChart, only 22% (4/18) of them became active users. Another 33% (6/18) had never heard of MyChart or was unsure of how to access it. Users primarily accessed test results and appointment schedules. The main facilitating factors for patient portal use were information and motivation from health care providers and concerns about pregnancy due to a history of miscarriage. Reported barriers were lack of educational resources, lack of care provider encouragement, and technical difficulties possibly exacerbated by low health literacy. Participants also suggested improvements for MyChart, especially the provision of discussion-based support for pregnant women.

**Conclusions:**

The one-time verbal introduction of MyChart does not meet current patients’ needs. Data reveal the need for more consistent patient education and support programs, tailored to patients’ previous pregnancy histories. The clinic also needs to facilitate better provider-patient communication about the importance of MyChart use.

## Introduction

### Patient Portal Use

A patient portal is a secure website through which patients can access their personal health information as a real-time, patient-centered record that makes information available instantly wherever and whenever it is needed [1]. The rates of patient portal adoption varied across different clinics among patients enrolled in AthenaHealth networks, with obstetrics and gynecology (OB/GYN) rates at 50% and pediatrics much lower at 23% [2]. Electronic patient portals are underutilized, particularly among low-income and minority populations [3-6]. A recent review reported that although 60% of patients enrolled in federally qualified community health centers registered with a patient portal system, only half of them used the account twice or more in 2 years [5], suggesting that patient portal activation does not necessarily lead to meaningful use among low-income populations.

### Digital Divide

A cohort study in a managed care organization found that about one third of patients registered with an available portal, and whites had higher rates of use than African Americans [7], leading the authors to confirm the existence of a digital divide and to argue that the expansion of patient portal use has the potential to widen disparities in health and health care. The digital divide may be caused or exacerbated by low health literacy [8,9]. Diabetes patients with self-reported limited health literacy had lower use of patient portal compared with those with adequate health literacy, independent of the effects of education and access to the internet. Considering the well-documented disparities in diabetes and other chronic diseases outcomes suffered by low-income populations, this digital divide in patient portal use may further exacerbate these trends. For that reason, disparities in patient portal use [3,10] were considered a barrier to meeting the Healthy People 2020 goals on health communication and health information technology [11]. A recent state of the science review on patient portals calls for future research focused on identifying specific populations and contextual considerations that would benefit most from patient portal use and analyzing the contextual factors that encourage or inhibit such use [12].

To date, there has been a lack of information and understanding of patient portal use among low-income pregnant women. It is known that a lack of patient-provider communication during prenatal visits and a lack of access to vital prenatal care information are risk factors for poor pregnancy outcomes [13,14]. Therefore, a patient portal may be an important technology-based health care communication venue to improve prenatal care delivery, especially in a rural area where mobile phone use is the norm, but access to physical health care is often limited due to a lack of transportation.

This exploratory study targeted low-income pregnant women and examined the reasons for and barriers to MyChart use, the characteristics of patients who became users, and the factors that patients suggested would increase their likelihood of use. These data provide an essential baseline for future MyChart patient education program design and implementation as well as for the development of prenatal clinic educational materials to be disseminated to improve patient adoption rates among low-income pregnant women.

## Methods

### Study Setting

The study site is an outpatient prenatal clinic affiliated with an academic medical center in a largely rural region characterized by high poverty rates in eastern North Carolina. *MyChart*, an Epic Systems Corporation (Verona, WI) patient portal, was introduced by this health system in October 2014. A nurse who was in charge of MyChart training program in the Center for Information Technology trained clinic staff once before MyChart implementation began. After a brief verbal introduction to MyChart, a nurse then followed a standard protocol to assist patients with enrolling in MyChart. There was no specific patient education program except for the distribution of an informational flyer with a helpline phone number and list of key features of the patient portal. Patients were given an activation code at their first appointment. They either had to then ask their health care provider (HCP) to activate the account while in the clinic or use the code themselves to activate the account from a home computer or mobile device. The activation code remained enabled for 6 months or until the patient declined MyChart use. After 6 months, the code expired and patients had to request a new one. Patients who made return visits to the clinic or any other HCP using MyChart were also offered new activation codes at those visits.

This study was undertaken as an exploratory sub-study of the ongoing Healthy Moms Study (HMS) being conducted by the authors. The HMS goal is to improve prenatal care by introducing a Facebook group as an intervention approach. To be eligible for the study, women had to be English-speaking, over the age of 18 years before the third trimester, and recipients of Medicaid insurance (an indicator of low-income status). A convenience sample of 24 women was invited to join the MyChart study during their HMS baseline assessment. Moreover, 4 patients dropped out, due to nonresponse (18% attrition rate), and 2 voice recordings were lost due to technical difficulties.

### Study Participants

The analytic sample of participants included 18 women, with the average age of 26.1 (20-37) years. Non-Hispanic, African American women accounted for 61.1% of participants. The average gestational age of mothers was 23.7 (SD 8.1) weeks pregnant at the time of recruitment, and 2 of the women were pregnant for the first time. In addition, 5 women were recruited from the high-risk clinic and 13 from the low-risk clinic. MyChart exposure time ranged between 3 and 41 weeks, with an average of 16.6 weeks among those who had activated their account during the observation period.

**MyChart interview questions.**

Have you ever used MyChart? (Why or why not)

(If used) How often do you interact with MyChart?

Do you find it useful? (Or do you think it will be useful?)

What is most useful about it? (What would make it more useful?)

What do you like most about MyChart?

What do you like least about MyChart?

Would you say that your health care providers motivate you to use MyChart?

When you have a question about something happening with your pregnancy, who do you talk to first? Is there a particular site you use for finding prenatal information?

Do you use any electronic social networking such as Facebook, Twitter, Instagram, or blogs that have provided you with information about your pregnancy?

Ad hoc questions: When you think about your life right now what are the three biggest worries that you have? What are your top three priorities?

### Data Collection and Analyses

A semi-structured interview assessed characteristics of MyChart users, perceptions and attitudes toward MyChart, and whether and how HCP encouraged MyChart use (Textbox 1). In addition, respondents were asked about sources consulted for prenatal advice and health information, and about participation in any prenatal support groups or classes, both online and offline. Interviews were conducted at the study clinic or over the phone at a later time between February and May 2015 before study participants enrolled in a Facebook intervention. The answers were digitally recorded and transcribed and checked by an investigator before being uploaded into N-Vivo (V.10; QSR International, Melbourne, Australia) for analysis. The East Carolina University Institutional Review Board approved this study.

MyChart themes were coded in the following categories by 2 coders: MyChart functions, improving MyChart, and participant-reported use of MyChart. These were divided into subcategories featuring functions and utilizations mentioned (easy access to health information, prescription refills, HCP communication, lab results, and appointments), reasons for using or not using MyChart, HCP motivations to use MyChart, and awareness of MyChart.

An electronic health records (EHR) review was conducted to collect information regarding age, race, pregnancy history, intent to breastfeed, as well as detailed information regarding MyChart activation and use, number of physical prenatal visits, and providers seen. After starting the study, the health literacy test and interview questions about life and pregnancy priorities and concerns were added to refine study aims as posthoc study measurements. The Newest Vital Sign Health Literacy Scale [15] was added after the study began to assess health literacy among 13 participants. Moreover, 7 mothers (53.8%) scored at the high level of adequate literacy, 4 (30.8%) scored at a moderate level of limited literacy, and 2 (15.4%) scored a low level of limited literacy. These scored groups were compared with rates of MyChart use.

Actual rates of MyChart activation and use were retrieved from the EHR review. Data included the date the portal was activated, declined, or expired and any communication between patient and HCP, viewing test results, number of physical visits, number of phone calls between patient and provider, and number of letters sent to the patient. By combining the interview and EHR data, participants were split into 4 MyChart *use* categories: *Active Users,* defined as those who activated their MyChart accounts and used them at least once before the end of the observation period; *Inactive Users,* those who activated their MyChart accounts but had not used them since activation; *Nonusers,* those who had heard of MyChart but had not activated their accounts; and *Unaware Users,* those who did not have a MyChart account and had never heard of MyChart or did not know how to access it.

Descriptive statistics were performed on the quantitative data because of the small sample size. The group differences in quantifiable information between MyChart use and pregnancy risk were tested by *t* test or Wilcoxon rank sum test depending on the distribution of the data (Table 1). The selected variables of prenatal care use are presented in Table 2.

## Results

### MyChart Users and Their Characteristics

Only 4 participants (22%) were active users, whereas 3 participants (17%) activated their account but did not use MyChart. Another 5 participants (33%) were unaware of MyChart or unsure how to access it (Table 1). None of the 5 nonusers (28%) activated their accounts. About 75% of active users reported that they logged in every time they received an email that test results were available, and one used MyChart to keep up with her health information after appointments. The inactive users had not logged in since activating their accounts.

**Table 1:** Participants’ characteristics by MyChart use.

Characteristics	Active users (n=4, 22%)	Inactive users (n=3, 17%)	Nonusers (n=5, 28%)	Unaware users (n=6, 33%)
Age, mean (SD)	27 (4.32)	23.3 (3.51)	25.8 (6.53)	25.5 (5.53)
Total parity, mean (SD)	0.5 (0.58)	0.66 (1.15)	2.6 (1.34)	2 (1.10)
African American, rate	50	100	60	50
Poor pregnancy history^a^, rate	100	0	80	83
Current pregnancy risk, high risk, rate	0	0	60	33

^a^Poor pregnancy includes miscarriage, ectopic pregnancy, and premature birth.

**Table 2:** The average number of prenatal care use by MyChart use and pregnancy risk.

Characteristics		Total physical visits	Doctor visits	Midwife visits	Ultrasound visits	Lab test visits	Phone calls	Letters to patient
**MyChart use, mean number (SD)**								
	Active users (n=4, 22%)	11.8	1.5	5.3	1.5	3.5	2.0	0.5
	Nonusers (n=5, 28%)	19.2^a^	8.6^b^	1.4^b^	4.0	5.2	6.4^c^	1.4
**Current pregnancy risk, mean number (SD)**									
	High risk (n=5, 28%)	17.8	8.4	0.8	4.6	4.0	13.8	1.6
	Low risk (n=13, 72%)	12.2^b^	2.0^b^	4.5^b^	1.7^b^	4.0	3.1^b^	0.3

^a^The mean differences between the 2 groups were detected at *P* value <.05 by *t* test or Wilcoxon rank sum test.

^b^*P*=.08.

^c^*P*=.08.

#### Women With a History of Poor Pregnancy Are More Likely to Use MyChart

According to EHR, 13 (72%) of the women had experienced poor pregnancy histories such as miscarriage, preterm birth, and ectopic pregnancy. All active users experienced miscarriage, and 3 of the nonusers had a preterm delivery (Table 1). One active user said she checked her account weekly and after every appointment, “to make sure I’m not missing anything.” For another participant, keeping track of her prenatal care was a top priority due to a previous miscarriage and a bad experience at a different clinic where she was misdiagnosed as ectopic. Another 4 participants (40%) who had miscarried previously were unaware of MyChart and 2 participants (20%) were nonusers. Two participants were pregnant for the first time and both were inactive users.

On average, nonusers had more physical visits than active users and made 7.1 times more physical visits to doctors (Table 2). Active users saw 3.9 times more midwives, suggesting a possible difference in the type of provider consulted.

#### MyChart Use Difference Between Low-Risk and High-Risk Pregnancies

Although 54% (7/13) of participants recruited from the low-risk clinic had activated their MyChart accounts, only 31% were active users. None of the patients recruited from the high-risk clinic were using MyChart (Table 1). All active users were in a low-risk pregnancy, although they had poor pregnancy histories such as previous miscarriages. Out of 13 low-risk patients, 8 had poor pregnancy histories. It is noted that all 5 patients recruited from a high-risk clinic had a poor pregnancy history, but none of them used MyChart. Patients seen in the high-risk clinic had, on average, 5.6 times more physical visits and saw 6.4 times more medical doctors than patients on the low-risk side of the clinic (Table 2). High-risk patients also had 10.7 times more phone calls than low-risk patients. Although slightly over half of the low-risk pregnancy participants used MyChart, none of the high-risk pregnancy participants used MyChart and they made more medical office visits.

#### Health Priorities and Concerns

In addition, 10 out of 11 mothers said their health or their baby’s health was a top priority and 6 out of 11 listed their health or their baby’s health as a top concern in their life situation. Other top concerns and priorities included financial concerns, looking for a job, parenting, and family. All 4 active users put either their baby’s or their health as a priority and 3 listed it as a top concern; the fourth listed her top concerns as related to finances, work, and family.

#### Technical Difficulties and Unknown Expiration Dates Are Barriers

MyChart activation was listed as pending for 5 participants (28%); 3 of these women had never heard of it and another did not know how to activate an account. Moreover, 4 women (22%) had actively declined to use MyChart, disabling their activation code, but 2 of these women claimed they had never heard of MyChart, indicating some possible miscommunication between providers and patients. One woman who had a MyChart account but did not use it said she never received a confirmation email and did not use it due to technical difficulties; however, the EHR lists her account as online and active, indicating there was no technical difficulty. Although the study clinic has a telephone hotline for MyChart problems, no one brought up the helpline in the interview.

#### Low Health Literacy Is a Possible Barrier

Health literacy was assessed among 13 of the 18 participants. All assessed active users scored at high or moderate levels of health literacy. Both participants who were low-level literacy were either in the inactive or unaware group. These limited results suggest that health literacy could potentially have an impact on MyChart use, with those scoring moderate to high being more likely to use MyChart when they are aware of the service.

### MyChart Features and Functions Among Users

#### Lab Test Results Are the Most Used Function

All of the active users mentioned test results as the key feature and all accessed their results. One of the inactive users mentioned that she thought it would be useful to check her lab results, but none of the nonusers mentioned the availability of test results. Medical records confirmed that MyChart was primarily used for tracking test results. Nonusers were more likely to mention wanting convenient access to health information, but few could name any specific features, and when asked how to improve MyChart, 2 responded suggesting services that are already available.

#### Scheduling Is the Second Most Popular Function

Active MyChart users mentioned appointment scheduling as well, with 3 users (75%) checking appointments. Only 1 nonuser (20%) mentioned access to appointment scheduling as a feature of MyChart, and 1 other nonuser thought that appointment information and scheduling would make her more likely to use the service, seemingly unaware that it was already available.

#### Communication With Health Care Providers

A total of 2 users (50%) mentioned the ability to communicate with their HCP online, but neither had used this service at the time of interview, although 2 had submitted feedback about their care through a patient satisfaction survey. Some respondents still mentioned communication with providers as a convenient option or *“* just-in-case *”* back up. One woman said:

It is super convenient…there is this little part where you can go and ask questions. I don’t know how quickly you get replies but I like that.

She later used this function to ask the nurse a question about taking iron supplements during pregnancy.

#### Prescription Refills and Medication Lists

Prescription refill was mentioned least often, with 1 nonuser admitting she had not used it yet, but thought that MyChart would be useful primarily for prescription refills and appointment scheduling. However, her activation code expired before she accessed these features. One active MyChart user mentioned the convenience of being able to access a list of her medications online but did not request refills.

### Improving MyChart

#### Health Care Providers Are the Strongest Facilitating Factor

A total of 7 women (39%) reported that their HCP did not motivate or encourage them to use MyChart, whereas 2 active users, 1 inactive user, and 1 nonuser said their HCP talked with them about MyChart but did not necessarily motivate them. One active user explained:

I feel like they had said something to me maybe once or twice about it…I guess it’s not really encouraged as much as they let you know that the option is there and they leave it up to you to decide.

Another said her HCP did *“* not usually *”* motivate her to use MyChart, but explained, *“* It’s always a different provider every time I go out there, which doesn’t make it any better. *”*

#### Lack of Patient Education and Inconsistent Provider Models Are the Main Barriers

Although MyChart users were mostly satisfied with the services provided, 2 of them (50%) thought it was not always easy to understand or access the information. One woman wanted clearer instructions and another found the information presented to be difficult to understand, which may relate to low health literacy. One inactive user and one unaware patient felt that if access to MyChart was easier or if they had better instructions, they would be more likely to use it. Other requests from nonusers included services they were not previously aware were available, such as doctor’s notes and appointment information.

#### Participants Use the Web for Prenatal Information But Not All Use MyChart

A total of 16 participants (89%) used the Web to look for prenatal information and advice and another 8 used social networking sites (SNS) to find prenatal information. Both the women who did not use the Web at all for prenatal information reported that they had never heard of MyChart. All active MyChart users used the Web to look for prenatal information and 75% of them used SNS. One inactive mother and 2 nonusers regularly used both the Web and SNS to look for prenatal information, indicating that access to the Web is not a barrier to MyChart use.

#### Participants Prefer to Seek and Share Information With Other Pregnant Women

A total of 14 (78%) of participants preferred to talk to other pregnant women or mothers or to read their comments and stories online over MyChart use. When asked how they verified information found online or where they went when they had a question about their pregnancy, 12 (66%) of participants said they talked with their HCP, whereas others said they would go on the internet or ask family or friends first. One MyChart user (20%) responded that she always talked to her doctor, whereas 3 others (75%) said they would talk with other mothers or check the internet first if it was a minor thing, then confirm with a doctor or nurse. Moreover, 4 nonusers (80%) turned to the internet first, then would confirm with a doctor, nurse, or more experienced mother.

#### Those Who Use Prenatal Support Are More Likely to Use MyChart

Half of active and half of inactive MyChart users were currently involved or planned to be involved in a face-to-face prenatal support group or class for their pregnancies. In addition, 2 (40%) of nonusers and 2 (33%) of unaware patients had been involved with a prenatal class or support group during a previous pregnancy. In addition, 69% of participants joined a Facebook group for prenatal support and education after receiving an invitation to do so, including 3 of the active MyChart users (75%). These findings suggest that mothers who proactively seek out either face-to-face or SNS prenatal support and educational opportunities may be more likely to use MyChart.

## Discussion

### Underused Patient Portal and Characteristics of Mothers Who Use Patient Portal

This is the first mixed-methods study to explore the use of and expectations about a patient portal among low-income pregnant women. We found that 39% (7/18) of participants interviewed signed up for MyChart, but only 22% (4/18) of participants were active users. Another 33% (6/18) had never heard of it or were unsure how to access or use it. This use rate is lower than previous studies for low-income populations [3,5] and lower than the OB/GYN clinics [2] but consistent with other general population surveys [12]. As patient portal adoption is one of the recommended measures of health care quality and safety [16], this study adds exploratory, qualitative information about some of the potential factors responsible for a digital divide among high- and low-income populations.

Among the patient characteristics of MyChart users, previous and current pregnancy problems were evaluated as patient portal adoption was higher among patients with high and moderate morbidity in the general population [9,17]. Our results are interesting; mothers who had poor pregnancy histories were more likely to use MyChart, but these women were all defined as low-risk patients during the current pregnancy at the time of the study. In contrast, women in high-risk pregnancies with previous poor pregnancy histories did not use MyChart. Although the sample size is very limited, we interpret this finding to reflect differences in communication preferences. Those women in current high-risk pregnancies made nearly twice as many medical office visits as low-risk patients. Therefore, they had more opportunities to ask questions and to receive specific health information face-to-face rather than relying on online means of health care communication as the low-risk patients did. Moreover, high-risk patients usually saw rotating physicians and residents rather than midwives, which is common in academic medical centers serving low-income populations. This lack of provider continuity and type may also contribute to inconsistent communications about the importance and use of the patient portal, which was one of the barriers women mentioned to MyChart use. As we did not measure care providers’ practices and attitudes about MyChart use based on pregnancy risk profile, it is not clear whether provider type (rotating HCP vs same HCP) or current and previous pregnancy risk best account for the difference in MyChart use.

The Stage 2 Meaningful Use requirements define patient adoption as downloading or viewing health information and communicating with a health care provider via secure messaging services [16], whereas other researchers use the initial sign up to define use, making direct comparisons among studies difficult. We confirmed that MyChart sign-up does not equal MyChart use. Although all active users in our study used MyChart to check test results, and 1 inactive user was anxious to do so, none of the nonusers mentioned this feature as an option. The perceived usefulness of available medical information and ease of site navigation played an important role in patient adoption and use [18]. Mothers who are online users are more likely to use MyChart. Almost every woman in the sample consulted online resources and several used SNS, similar to other reports of high Web use among pregnant women. Even the sources used to verify prenatal information varied [19]; we found that mothers who use SNS, including Facebook, blogs, discussion boards, and chat sites, were more likely to use or be interested in MyChart and to want to join a Facebook group designed for pregnant women only. Due to the small sample size, it is difficult to generalize, but low level of health literacy seems to be a systematic barrier as both patients with low literacy did not use MyChart. Limited health literacy may affect patients’ abilities to activate and navigate the particular features of MyChart, which involve more technical and knowledge-based literacy than SNS sites that draw on personal experience and opinion sharing. A recent qualitative study reported that limited health literacy seems to be a fundamental barrier among low-income patients and caregivers with chronic disease [20].

### Lack of Patient Education and Communication in Prenatal Health Care

The accuracy of internet information was judged based on relevance to the participant’s own symptoms and condition. Instead of using dry but informative articles, these women preferred to read about the experiences of other pregnant women. In their eyes, this information was *“* more accurate *”* because it was more relevant to them. This preference should be expected to carry over to MyChart use as it is an extension of internet engagement, allowing women to track their test results and appointments and to talk to their HCP if needed. Yet, few had actually used MyChart to communicate with providers, and the portal does not provide much patient-friendly information and support. These findings suggest that a redesign of key features and more active participation by HCP’s might make MyChart more attractive to pregnant women.

The communication style of care providers with patients played an important role in enhancing patients' self-care behaviors among diabetics [21] and those with chronic disease, including their assessments of subjective health status [22,23]. Prior studies consistently reported that the use of patient portal systems enhanced patient-provider communication [24,25], decreased missed appointments among traditionally disadvantaged groups [12], and had the potential to improve health or medication adherence [26]. Our study supports that provider interaction seems to have a positive effect on MyChart use [12], as those who were prompted by their HCP to use MyChart were more likely to have activated their accounts. Lack of patient education about MyChart—what it was, what services it offered, and how to use it—is the most common barrier across the MyChart user groups. One of the nonusers said *:*

I haven’t asked anybody how to do it…they mention it but I haven’t heard anything else about it. They were just saying that you can go on MyChart and look up your information

One nonuser thought it would be helpful if appointment information was included, and another thought she would be more likely to use it if the pictures and notes from ultrasounds were posted. Appointment information is available through MyChart, and ultrasound photos are not always posted. The notes are available through MyChart as well, indicating a lack of patient education and miscommunication or misunderstanding of MyChart function.

This study has limitations. We did not assess health care providers’ experiences and attitudes about MyChart use or their compliance rates with MyChart introduction protocols. The small sample size and relatively short period of observation time for MyChart use limited the interpretation of the results. Especially, the noted group differences by descriptive statistical tests may be due to chance only. Moreover, we did not ask how patients understand and consider offline visits in comparison with communication through MyChart. Access to the internet might be an important barrier to MyChart adoption. However, a majority of the women in our study reported seeking prenatal health information online. Furthermore, data from a previously conducted needs assessment at the study clinic found that only 1 patient (1%) out of 86 pregnant women lacked access to the internet on a regular basis. Thus, internet access does not appear to be the critical barrier for today’s low-income pregnant women.

In conclusion, low-income mothers relied on general internet sites and the advice of family and friends for their prenatal health information instead of the secure online patient portal offered by their HCP. Although most patients who did use the portal reported satisfaction with MyChart’s features, some found the information difficult to understand, and users and nonusers alike wished for clearer instructions to understand lab test results. In addition, these data show that low-income mothers want more than factual information delivered electronically. They indicated a clear preference for a discussion place, either on SNS or in person, so they could share and learn from other mothers who experiencing similar health issues, which suggests the need to redesign the patient portal to incorporate more diverse functions. The findings also suggest that pregnant women with previous poor pregnancy histories may be more likely to use patient portals and to engage in monitoring their prenatal health status. In addition, in-depth research is warranted to examine more systematically the dynamics of patient portal use among pregnant women enrolled in a high-risk clinic who are already experiencing a large number of office visits. Finally, there seems to be a gap between patients and providers in health communication with MyChart. Future research should focus on the best ways to provide a patient-oriented communication channel via patient portals that fits a group care model.

## Abbreviations

EHR: electronic health record
HCP: health care provider
HMS: Healthy Moms Study
OB/GYN: obstetrics and gynecology
SNS: social networking sites
